# Multi-phase synthetic contrast enhancement in interventional computed tomography for guiding renal cryotherapy

**DOI:** 10.1007/s11548-023-02843-z

**Published:** 2023-02-15

**Authors:** Mark A. Pinnock, Yipeng Hu, Steve Bandula, Dean C. Barratt

**Affiliations:** 1 Centre for Medical Image Computing, University College London, London, UK; 2 Wellcome/EPSRC Centre for Interventional and Surgical Sciences, University College London, London, UK; 3 Centre for Medical Imaging, Division of Medicine, University College London, London, UK; 4 Department of Interventional Radiology, University College London Hospitals NHS Foundation Trust, London, UK

**Keywords:** Computed tomography, Contrast enhancement, Convolutional neural network, Interventional radiology, Deep learning

## Abstract

****Purpose**:**

Minimally invasive treatments for renal carcinoma offer a low rate of complications and quick recovery. One drawback of the use of computed tomography (CT) for needle guidance is the use of iodinated contrast agents, which require an increased X-ray dose and can potentially cause adverse reactions. The purpose of this work is to generalise the problem of synthetic contrast enhancement to allow the generation of multiple phases on non-contrast CT data from a real-world, clinical dataset without training multiple convolutional neural networks.

****Methods**:**

A framework for switching between contrast phases by conditioning the network on the phase information is proposed and compared with separately trained networks. We then examine how the degree of supervision affects the generated contrast by evaluating three established architectures: U-Net (fully supervised), Pix2Pix (adversarial with supervision), and CycleGAN (fully adversarial).

****Results**:**

We demonstrate that there is no performance loss when testing the proposed method against separately trained networks. Of the training paradigms investigated, the fully adversarial CycleGAN performs the worst, while the fully supervised U-Net generates more realistic voxel intensities and performed better than Pix2Pix in generating contrast images for use in a downstream segmentation task. Lastly, two models are shown to generalise to intra-procedural data not seen during the training process, also enhancing features such as needles and ice balls relevant to interventional radiological procedures.

****Conclusion**:**

The proposed contrast switching framework is a feasible option for generating multiple contrast phases without the overhead of training multiple neural networks, while also being robust towards unseen data and enhancing contrast in features relevant to clinical practice.

## Introduction

Minimally invasive, image-guided treatment options for renal cell carcinoma, such as cryoablation, offer a lower complication rate and a faster recovery period than radical or partial nephrectomy [1] by using needles to cause tumour cell death via extreme cold [2]. Interventional computed tomography (iCT) offers excellent 3-dimensional visualisation of the relevant anatomy [3], but requires intravenous iodinated radiocontrast agents (RCAs) to improve soft tissue contrast. RCAs washout quickly requiring repeat administration and an increase in X-ray dose to the patient [4], can trigger allergic reactions and cause renal toxicity [5]. Adequacy of contrast enhancement also varies significantly between patients, with some image volumes exhibiting inadequate contrast even with the use of automatic bolus tracking [6].

To reduce usage of RCAs, it is necessary to develop computational techniques that can correctly alter the contrast of the clinically relevant regions of interest (ROIs). Histogram-based methods [7] modify the overall image contrast but cannot preferentially enhance specific regions. Blood vessels can be enhanced using level set techniques [8] and banks of oriented filters [9], but these do not generalise well to other ROIs. Convolutional neural networks (CNNs) are ubiquitous in medical imaging and are now being used for reduction of gadolinium dosage in neuroimaging [10–12], improving liver tumour contrast [13], converting contrast-enhanced (CE) CT to non-contrast-enhanced CT (NCE) [14–16] and improving magnetic resonance flow imaging contrast [17].

Creating synthetic contrast-enhanced (sCE) CT images without RCAs has been proposed [18, 19], using both a coarse-to-fine generative adversarial network (GAN) training strategy [20] and using CycleGANs on unpaired data [21, 22]. While these studies have shown sCE to be feasible, they focus on the early arterial phase, and use data with a simple mapping from NCE to CE (acquired close together in time) whereas the ability to generalise to iCT images (where the mapping from input images to ground truth is more complex) is unknown. In our institution, the pre-procedural imaging in a typical cryoablation procedure involves a NCE series, followed by two CE series at 35 and 80 s after injection of RCA. These correspond to corticomedullary (CME) and nephrogenic enhancement (NGE) phases, after which the enhancement fades as RCA is eliminated by renal filtration [23]. It would be advantageous to generate multiple contrast phases—for instance, NGE images offer high sensitivity for the detection of renal tumours, while lower false-positive rates are possible with CME images [24]. While this could be accomplished using a CNN trained for each phase, a network conditioned on phase information could perform the same task on multiple time points in the procedure without training additional networks.

To enforce quantitatively realistic intensities for performing clinically relevant downstream tasks, for instance, augmenting scarce data, enhancing images prior to segmentation, RCA dose reduction or identifying tumours in interventional procedures, the distribution of the sCE images should be as close to the actual CE data distribution as possible. An evaluation framework needs to take into account not only image quality and predicted intensities, but also the bias of a given technique compared to the ground truth.

Adversarial training has become increasingly popular in the super-resolution and style transfer literature, as supervised learning techniques using the $L^1$- or $L^2$-norm can lead to excessive blurring. Pix2Pix [25] was developed for image-to-image translation, where the $L^1$-norm between the source (NCE) and target (CE) images acts as a supervisory signal constraining the generator’s output to be close to the real target. As this requires paired data, CycleGAN [26] was introduced as an unpaired alternative, using two generators and two discriminators, one pair of which maps images from the source domain to the target domain, while the other pair performs the reverse operation. This process is regularised by the *cycle-consistency* term, which enforces a consistent forward and backward mapping between the two domains but does not prevent the generator from removing (or adding) features that may (or may not) be present in the target image [27]. In medical imaging applications, this is highly undesirable; inaccurate predictions may lead to incorrect diagnoses or inaccuracies in image-guided procedures. The additional advantage of supervision for contrast transfer between NCE and CE medical images is that the training process can theoretically benefit from the ground truth intensities of the contrast-enhancing ROIs.

The aim of this work is twofold. First, as a step towards accurate, clinically useful, multi-phase sCE we propose a framework for contrast phase switching and test it against separately trained networks as a baseline. Second, we comprehensively evaluate the quantitative performance of the aforementioned training paradigms (ranging from unpaired adversarial to fully supervised training) on contrast transfer between NCE and CE images.

To the best of our knowledge, this is the first study performing sCE on interventional CT data that pose challenges such as organ displacement and needle insertion. While iCT data are scarce, we show significance on a real-world dataset that is the largest we have found so far in the literature. In addition, we believe it is the first attempt to generate synthetic contrast conditioned on phase information, allowing the user to readily switch between phases during inference. As sCE is a new interventional application, we focus on validation and reporting first quantitative results using multiple established neural network architectures in the style transfer domain, leaving the development of new models specific to sCE to future work. The research questions posed are:

1. Does the proposed multi-contrast sCE framework match the performance of individually trained CNNs?
2. Does a supervisory signal improve performance in sCE compared to unsupervised adversarial training?
3. Are the generated intensities quantitatively realistic and of adequate quality to allow their use in clinically relevant tasks?

To answer the above questions, we have made the following contributions: (1) We use deep learning to perform multi-phase sCE on pre-procedural iCT images; (2) We compare three training paradigms on this task, exploring whether supervision improves model accuracy; (3). We use Bland–Altman analysis to quantitatively measure the bias of the proposed methods, showing that they generate more consistent enhancement than real CE, and that a network’s bias can affect its performance on downstream tasks; (4). We examine whether these contributions aid a U-Net [28] in segmenting tumours in sCE images; (5). Lastly, we assess the two most promising models on out-of-distribution intra- and post-procedural data and show that they maintain their performance while improving visualisation of previously unseen high-contrast features such as needles. This shows promise for improving visualisation of features encountered in interventional radiology procedures, for instance, needles as well as the ice ball formed during the cryotherapy process.

## Methods

### Contrast switching

The proposed method for allowing contrast phase switching in generated images can be applied to any CNN and is achieved by tiling a scalar quantity representing the required phase (1 for CME, 2 for NGE), which is then concatenated to the feature maps in a layer. A standard CNN $f_{\varvec{\theta },t}$ trained for a particular phase *t* maps a source image ${\mathbf {x}}$ to the appropriate target $\hat{{\mathbf {y}}}_t$:

$$\begin{aligned} \hat{{\mathbf {y}}}_t = f_{\varvec{\theta },t} \, ({\mathbf {x}}) \end{aligned}$$

The phase switching network $f_{\varvec{\theta }}$ instead takes the phase information as an input:

$$\begin{aligned} \hat{{\mathbf {y}}}_t = f_{\varvec{\theta }} \, ({\mathbf {x}},t) \end{aligned}$$

Three classes of machine learning methods are described as follows to implement this phase-conditioned contrast prediction task, chosen for being well-validated methods in the style transfer literature that encompass the full range of supervision, from fully supervised to fully adversarial. As these architectures are well established in the literature, we omit the details for brevity and refer the reader to the specific papers, focusing instead on the modifications to achieve the aims set out above.

### Fully supervised

The baseline technique (UNet-Baseline) comprises two 3D U-Nets [29], trained separately on CME and NGE images, while the phase switching network (UNet-Phase) incorporates the phase information as described in each layer of the decoder except the output layer. Each down-sampling block in the encoder uses one convolutional layer, while up-sampling decoder blocks employ a transpose convolution and then a standard convolution after concatenation with the skip layer. Both implementations employ instance normalisation, commonly used in style transfer applications [30]. The fully supervised loss ${\mathcal {L}}_{ROI}$ is the mean absolute error. To improve convergence, we split the objective into foreground ${\mathcal {L}}_F$ and background ${\mathcal {L}}_B$ losses, weighted by a hyper-parameter $\mu$, where the foreground consists of arterial, kidney and tumour ROIs extracted by a mask (represented by the indicator function ${\mathbf {1}}$ for foreground ${\mathbf {1}}_F$ and background ${\mathbf {1}}_B$):

$$\begin{aligned}&{\mathcal {L}}_F = \Vert {\varvec{1}}_F \, [{\varvec{y}}_t - f_{\varvec{\theta }} \, ({\varvec{x}}, t)] \Vert _1\\&{\mathcal {L}}_B = \Vert {\varvec{1}}_B \, [{\varvec{y}}_t - f_{\varvec{\theta }} \, ({\varvec{x}}, t)] \Vert _1\\&{\mathcal {L}}_{ROI} = \, \mu {\mathcal {L}}_F + (1 - \mu ) \, {\mathcal {L}}_B\\ \end{aligned}$$

### Adversarial with supervision

**Fig. 1:**
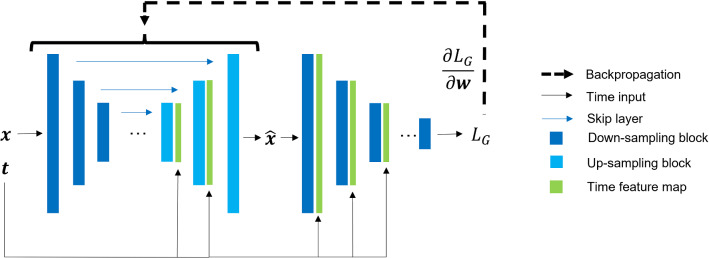
Training pipeline for Pix2Pix generator

The network used for adversarial training with supervision is based on the Pix2Pix architecture [25]. The generator $f_{\varvec{\theta }}$ is a U-Net identical to that described above, while the discriminator $g_{\varvec{\phi }}$ is a 3D version of the one described in the Pix2Pix paper, and noise $\mathbf{z }$ is added to the generator using 50% dropout in the first three layers of the decoder both during training and at inference. Phase information was added to both the generator decoder (identically to UNet-Phase) and discriminator as in Fig. 1.

The Pix2Pix training objective uses the adversarial binary cross-entropy loss for discriminator and generator (${\mathcal {L}}_D$ and ${\mathcal {L}}_G$) below and reuses the fully supervised loss ${\mathcal {L}}_{ROI}$ as the supervising term for the generator, weighted by a hyper-parameter $\lambda$. The expectations are taken over the target images $\mathbf{y }$ and the source images with noise $\mathbf{x }, \mathbf{z }$.

$$\begin{aligned} {\mathcal {L}}_D&= -{\mathbb {E}}_{{\varvec{y}}}\{\ln \,[g_{\varvec{\phi }}({\varvec{y}}_t, t)]\} - {\mathbb {E}}_{{\varvec{x}}, {\varvec{z}}}\{\ln \,[1 - g_{\varvec{\phi }}(f_{\varvec{\theta }}({\varvec{x}}, {\varvec{z}}, t), t)]\}\\ {\mathcal {L}}_G&= -{\mathbb {E}}_{{\varvec{x}}, {\varvec{z}}}\{\ln \,[g_{\varvec{\phi }}(f_{\varvec{\theta }}({\varvec{x}}, {\varvec{z}}, t), t)]\} + \lambda \, {\mathcal {L}}_{ROI}\\ \end{aligned}$$

### Unpaired adversarial

The unsupervised method for comparison is CycleGAN [26]. Unlike the original CycleGAN implementation for unpaired images, we retain the U-Net-style Pix2Pix generator above (as in Xie et al. [22]) as the skip connections can propagate high-frequency information between paired source and target images. This allows us to isolate the effect of different degrees of supervision on the same generator architecture.

The loss comprises two least square adversarial terms for the forward mapping networks $f_{\varvec{\theta }}$, $g_{\varvec{\phi }}$ with parameters $\varvec{\theta }$ and $\varvec{\phi }$ (shown below) and two for the backward networks $f_{\varvec{\theta '}}^{-1}$, $g_{\varvec{\phi '}}^{-1}$ with parameters $\varvec{\theta '}$ and $\varvec{\phi '}$, along with the cycle-consistency loss ${\mathcal {L}}_{cyc}$. An additional identity loss ${\mathcal {L}}_I$ constrains the overall image intensities to be similar between source and target.

$$\begin{aligned} {\mathcal {L}}_D&= -{\mathbb {E}}_{{\varvec{y}}}[(g_{\varvec{\phi }}({\varvec{y}}_t, t) - 1)^2] - {\mathbb {E}}_{{\varvec{x}},{\varvec{z}}}[(g_{\varvec{\phi }}(f_{\varvec{\theta }}({\varvec{x}}, {\varvec{z}}, t), t)^2]\\ {\mathcal {L}}_G&= -{\mathbb {E}}_{{\varvec{x}}, {\varvec{z}}}[(g_{\varvec{\phi }}(f_{\varvec{\theta }}({\varvec{x}}, {\varvec{z}}, t), t) - 1)^2] + \lambda \, {\mathcal {L}}_{cyc} + \frac{\lambda }{2}\, {\mathcal {L}}_I\\ \end{aligned}$$

where

$$\begin{aligned}&{\mathcal {L}}_{cyc} = \Vert {\varvec{x}} - f_{\varvec{\theta '}}^{-1}(f_{\varvec{\theta }}({\varvec{x}}, {\varvec{z}}, t)) \Vert _1 + \Vert {\varvec{y}}_t - f_{\varvec{\theta }}(f_{\varvec{\theta '}}^{-1}({\varvec{y}}_t, {\varvec{z}}, t)) \Vert _1\\&{\mathcal {L}}_I = \Vert {\varvec{x}} - f_{\varvec{\theta '}}^{-1}({\varvec{x}}, {\varvec{z}}, t) \Vert _1 + \Vert {\varvec{y}}_t - f_{\varvec{\theta }}({\varvec{y}}_t, {\varvec{z}}, t) \Vert _1 \end{aligned}$$

**Table 1:** Image quality

Model	NCE $\rightarrow$ CME	NCE $\rightarrow$ NGE
MSE		
UNet-Baseline	7958 [6978, 9263]	7813 [7046, 9796]
UNet-Phase	8401 [7657, 11458]	8484 [7921, 10862]
Pix2Pix	8374 [7190, 9713]	8011 [6390, 10172]
CycleGAN	9612 [8102, 12031]	9218 [8194, 11655]
pSNR		
UNet-Baseline	57.32 [55.85, 57.83]	57.40 [5.46, 57.81]
UNet-Phase	57.09 [55.12, 57.45]	57.04 [55.62, 57.35]
Pix2Pix	57.10 [56.58, 57.67]	57.29 [55.93, 58.09]
CycleGAN	56.50 [55.24, 57.14]	56.68 [55.37, 57.14]
SSIM		
UNet-Baseline	0.9946 [0.9933, 0.9957]	0.9949 [0.9926, 0.9961]
UNet-Phase	0.9943 [0.9920, 0.9958]	0.9936 [0.9904, 0.9941]
Pix2Pix	0.9937 [0.9924, 0.9953]	0.9941 [0.9921, 0.9958]
CycleGAN	0.9940 [0.9928, 0.9965]	0.9940 [0.9918, 0.9957]

Data format: median [95% CI]

### Statistical analysis

When testing hypotheses in normally distributed data, the two-tailed t-test was used, while the Kruskal–Wallis H-test and Mann–Whitney U-test (also two-tailed) were employed for non-normal distributions. Confidence intervals for non-normal data were generated with the nonparametric bootstrap using 100,000 runs.

To assess the bias between the proposed techniques and the ground truth ROI intensities, Bland–Altman analysis [31] is used. This quantifies the bias between a measurement technique and a gold standard reference. The mean ${\overline{I}}$ of the predicted and ground truth intensities for each subject are plotted against the difference between the predicted values and ground truth $\Delta I$. The relationship between ${\overline{I}}$ and $\Delta I$ describes the bias, while the 95% limits of agreement (LoA) are 1.96 standard deviations around this bias. Linear regression distinguishes between constant bias (significant intercept with non-significant slope on t-test) and bias that changes proportionally with ${\overline{I}}$ (significant slope).

To compare biases between the techniques, the general linear model is used with ANCOVA and interaction terms. After testing interaction term significance to ensure no difference between bias slopes, ANCOVA tests for significantly different mean biases between techniques, controlling for intensity as a covariate, which may otherwise affect the mean bias in models that vary proportionally with intensity. This can allow comparison of models trained, for instance, in different institutions with different datasets. Pairwise statistical contrast analysis [32] is then performed to determine which models are significantly different from one another. All *p*-values from statistical tests underwent the Bonferroni correction for multiple comparisons as needed to maintain a family-wise error rate ($\alpha$) of 0.05.

## Experiments

### Data and pre-processing

The retrospective data were fully anonymised after approval from the local clinical governance committee and comprised images from renal cryoablation procedures performed by the interventional oncology service at University College London Hospital. The target labels were CME and NGE phase scans, while the source data were NCE scans, downsampled to a resolution of $256 \times 256$ with variable depth. Image intensity values were windowed to [$-500$, 2500] to remove noise and outliers before normalisation to [0, 1]. For the foreground mask, aorta and major tributaries, kidneys and tumour were segmented from the CE volumes in 3D Slicer [33]. The training set consisted of 35 procedures (34 subjects), of which 5 subjects were randomly selected for use in hyper-parameter tuning, while the test dataset comprised 15 subjects. Patch-based techniques have become common-place in the super-resolution literature, and have been proposed to preserve geometrical structure in style transfer applications [34]—we therefore train all networks on $64 \times 64 \times 64$ patches. The full-sized images are generated by running the model over the input image with stride 16 and averaging the output from overlapping patches.

### Network training

The networks were trained using Tensorflow 2.3 on a Nvidia P5000 16Gb graphics card with minibatch size 1, Adam optimiser ($\beta _1 = 0.5$, $\beta _2 = 0.999$) and He initialisation. The CycleGAN discriminators were trained using a buffer of 50 previous generated images as in the original paper, while a differentiable data augmentation strategy [35] using random translation, cut-out and intensity/contrast alteration was applied to both real images and generated images seen by the discriminator in Pix2Pix and CycleGAN. The fully supervised U-Nets employed a standard data augmentation routine using rotation, flipping, scaling, shearing and translation.

### Evaluation

All predicted images were converted back to Hounsfield Units (HU) before evaluation with visual examination, mean squared error (MSE), peak signal-to-noise ratio (pSNR) and structural similarity index measure (SSIM). For quantitative comparisons, five equally spaced slices of aorta (*Ao*), renal cortex (*Co*), medulla (*Md*) were segmented, as well as the entire tumour (*Tu*) except in one subject where the tumour could not be confidently identified. The ROIs are referred to by their abbreviations with the phase as a subscript, e.g. $Co_{CME}$ for cortex CME phase and $Ao_{NGE}$ for aorta NGE phase.

To determine whether the sCE images could be used for segmentation, U-Nets [28] were trained to segment 2D $64 \times 64$ patches from each of the evaluated methods using a minibatch of size 64 and learning rate $10^{-3}$. The training dataset comprised slices from each model’s predicted images for 10 of the 14 test subjects with an identifiable tumour (247 total) as well as the ground truth CE images (241 total). For testing, slices from the 4 remaining test subjects were used, totalling 91 for the model output and 95 for ground truth.

### Hyperparameter tuning

The hyperparameters tuned were the learning rates, number of layers, number of channels, $\lambda$ and $\mu$. All discriminators used 16 first layer channels and 3 layers, while the generators had 32 channels and 5 layers. The U-Net strategy used a learning rate of $1.0 \times 10^{-4}$ and $\mu$ of 0.2, while the CycleGAN strategy used learning rates of $2.0 \times 10^{-4}$ and kept the default cycle consistency weight of 10 and identity loss of 5. The Pix2Pix had discriminator and generator learning weights of $2.0 \times 10^{-5}$ and $3.5 \times 10^{-4}$, with $\lambda$ of 720 and $\mu$ of 0.1.

## Results and discussion

**Fig. 2:**
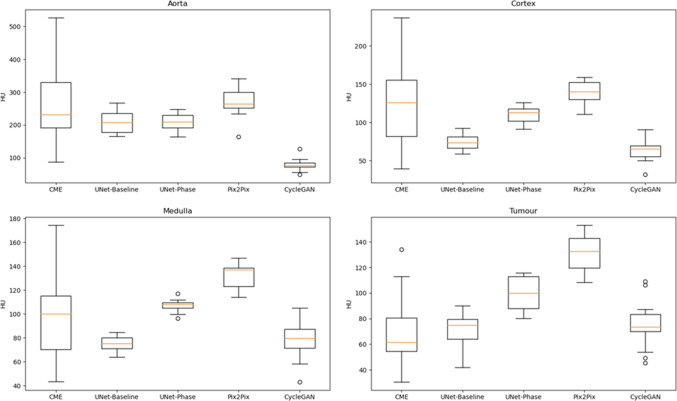
ROI intensities for generated CME images

**Fig. 3:**
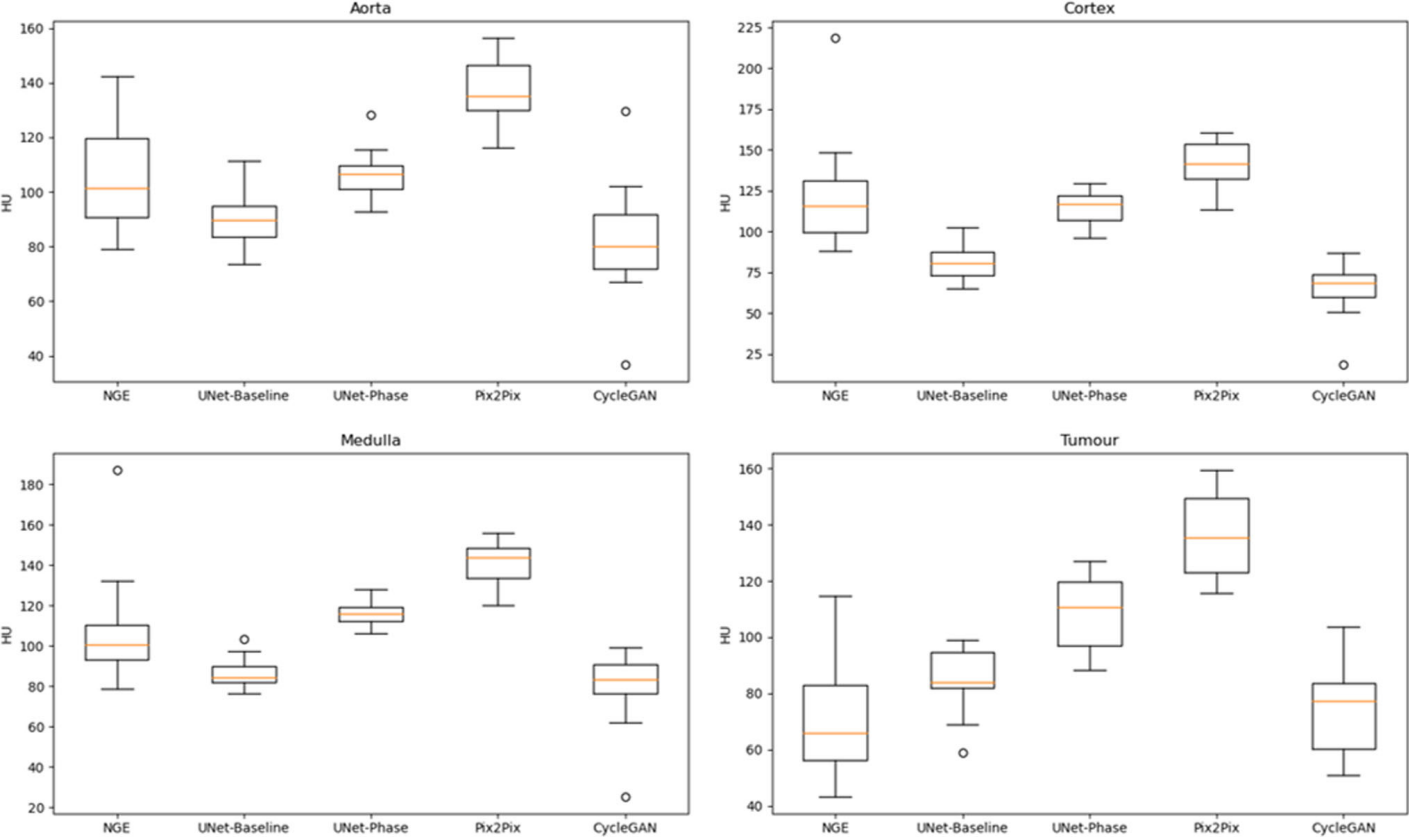
ROI intensities for generated NGE images

**Fig. 4:**
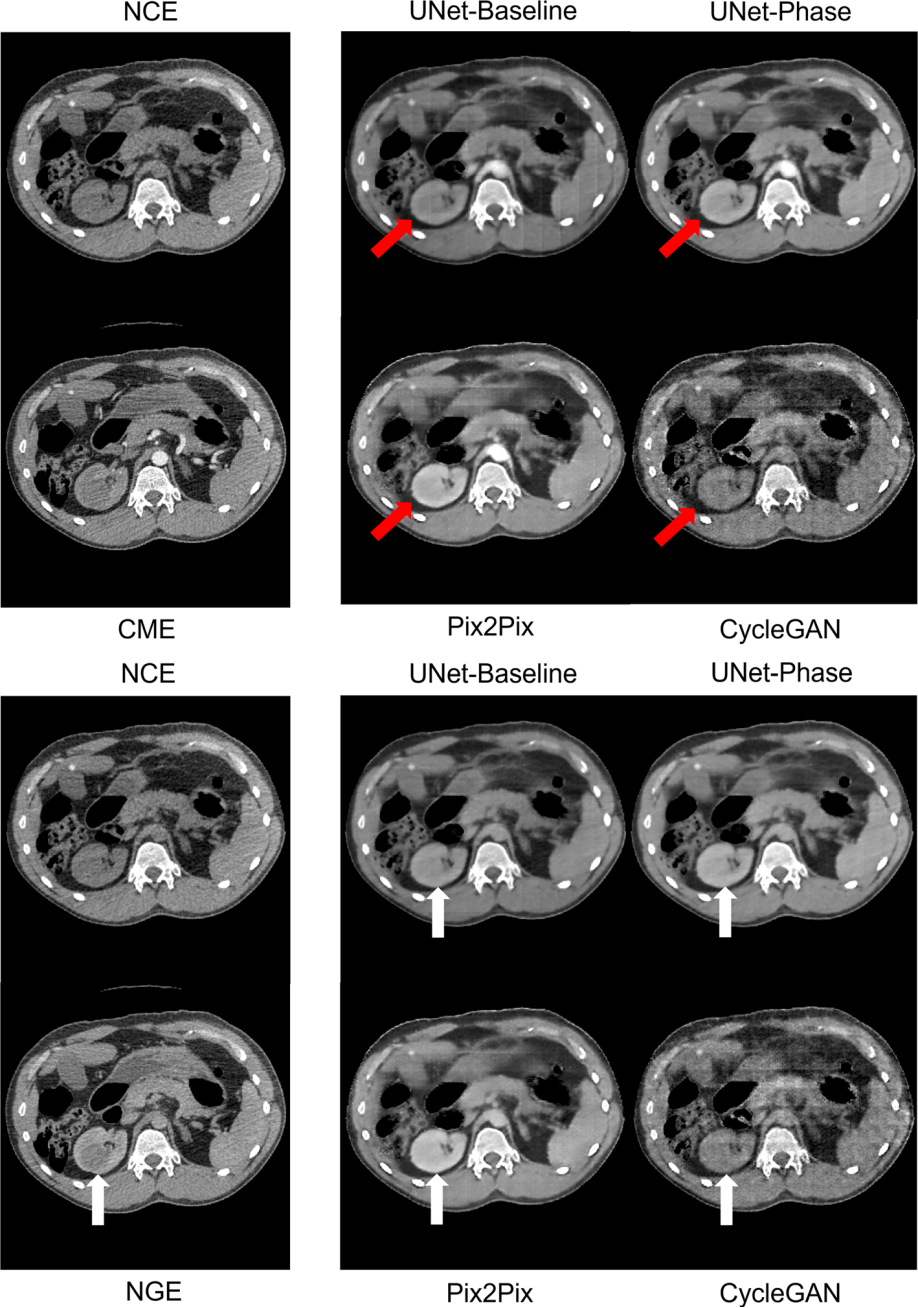
Example predictions for corticomedullary contrast (top block) and nephrogenic contrast (bottom block). Red arrows: cortical enhancement, white arrows: poor tumour contrast

**Fig. 5:**
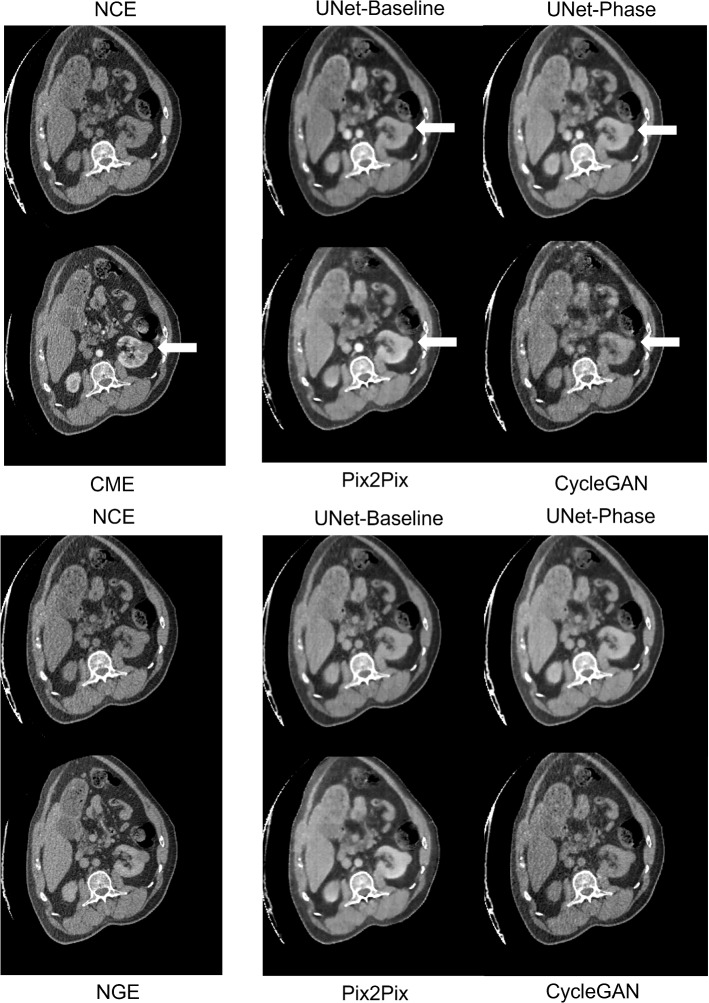
Example predictions for corticomedullary contrast (top block) and nephrogenic contrast (bottom block). White arrows: poor tumour contrast

Table 1 shows image quality metrics with confidence intervals generated via bootstrap. Figures 2 and 3 show the intensities generated by each network for each ROI, along with the ground truth intensities. Table 2 shows the mean and proportional bias between predicted intensities and ground truth for each of the models, as derived from the ANCOVA intercept term and slope, respectively, while Figs. 4 and 5 show examples from each network for both phases (Table 3).

**Table 2:** ROI biases and slopes

Model	CME Bias (HU)	NGE Bias (HU)
Aorta		
UNet-Baseline	$-48 \, [\pm 105]\, (\mathbf {-1.39})$	$-15\, [\pm 29]\, (-0.98)$
UNet-Phase	$\mathbf {-47}\, [\pm 74]\, (\mathbf {-1.45})$	$1\, [\pm 27]\, (-1.19)$
Pix2Pix	$14 \,[\pm 119]\, (\mathbf {-1.16})$	$\mathbf {30}\, [\pm 25]\, (-0.57)$
CycleGAN	$\mathbf {-178}\, [\pm 67]\, (\mathbf {-1.95})$	$\mathbf {-24}\, [\pm 38]\, (0.05)$
Cortex		
UNet-Baseline	$\mathbf {-50}\, [\pm 39]\, (\mathbf {-1.86})$	$\mathbf {-39}\, [\pm 36]\, (\mathbf {-1.59})$
UNet-Phase	$-15\, [\pm 39]\, (\mathbf {-1.84})$	$-6\, [\pm 38]\, (\mathbf {-1.50})$
Pix2Pix	$15\, [\pm 52]\, (\mathbf {-1.51})$	$21\, [\pm 46]\, (-1.08)$
CycleGAN	$\mathbf {-62}\, [\pm 51]\, (\mathbf {-1.78})$	$\mathbf {-55}\, [\pm 45]\, (-0.93)$
Medulla		
UNet-Baseline	$\mathbf {-25}\, [\pm 23]\, (\mathbf {-1.79})$	$\mathbf {-21}\, [\pm 26]\, (\mathbf {-1.60})$
UNet-Phase	$7\, [\pm 18]\, (\mathbf {-1.83})$	$9 \,[\pm 19]\, (\mathbf {-1.59})$
Pix2Pix	$\mathbf {31}\, [\pm 33]\, (\mathbf {-1.48})$	$\mathbf {33}\, [\pm 31]\, (-1.10)$
CycleGAN	$-22 \,[\pm 56]\, (\mathbf {-1.66})$	$-27\, [\pm 51]\, (-0.62)$
Tumour		
UNet-Baseline	$1\, [\pm 43]\, (-1.40)$	$13\, [\pm 41]\, (-1.32)$
UNet-Phase	$\mathbf {29}\, [\pm 44]\, (-1.23)$	$\mathbf {37}\, [\pm 45]\, (-0.87)$
Pix2Pix	$\mathbf {61}\, [\pm 45]\, (-1.05)$	$\mathbf {66}\, [\pm 47]\, (-0.55)$
CycleGAN	$5\, [\pm 61]\, (-0.85)$	$3\, [\pm 51]\, (-0.40)$

Data format: mean bias [95% LoA] (slope coefficient)

**Bold** indicates a significant proportional or constant bias for slope coefficient and mean bias, respectively

**Table 3:** Dice scores

Model	CME	NGE
Ground truth	0.44 [0.27, 0.62]	0.65 [0.59, 0.81]
UNet-Baseline	0.43 [0.36, 0.49]	0.43 [0.34, 0.50]
UNet-Phase	0.50 [0.43, 0.59]$^*$	0.44 [0.36, 0.60]
Pix2Pix	0.37 [0.28, 0.46]$^*\dagger$	0.29 [0.06, 0.38]
CycleGAN	0.49 [0.41, 0.54]$\dagger$	0.50 [0.44, 0.57]

Data format: median [95% CI]

$^*$ and $\dagger$ indicate significant difference ($p < 0.01$)

### Separately trained networks versus phase conditioning

The first hypothesis to be tested was whether training one network to switch between two contrast phases (UNet-Phase) was feasible compared to training one network for each phase (UNet-Baseline). There was no significant difference on H-test between any of the techniques in terms of image quality metrics (Table 1), and no discernible differences in image quality between UNet-Baseline and UNet-Phase on visual inspection (Figs. 4 and 5). UNet-Baseline significantly ($p < 0.001$) under-enhanced both cortical and medullary ROIs for both phases (Table 2), while UNet-Phase over-enhanced tumour ($p < 0.001$) for both phases as well as under-enhancing $Ao_{CME}$. The visual difference in cortical/medullary enhancement is visible in Fig. 4 (red arrows).

This demonstrates that it is possible to achieve phase-switching in most ROIs (with superior performance in kidney), opening up the possibility of generating multiple contrast phases later in the procedure when a lesion can be more conspicuous [23].

### Effect of supervision

The second hypothesis concerned the most suitable training paradigm for contrast enhancement, specifically that a supervisory signal would constrain the network to output realistic intensities.

The poorest performance was from CycleGAN (entirely unsupervised) in terms of visual appearance, despite no statistical significance in terms of image quality metrics (Table 1). CycleGAN under-enhanced aortic and cortical ROIs in both phases ($p < 0.001$, Table 2)—correspondingly, there is little discernible enhancement in Figs. 4 and 5. CycleGAN’s poor performance could be attributed to the lack of a supervisory term containing ground truth contrast information. In addition, the identity term ${\mathcal {L}}_I$ in the loss constrains the generator to leave unchanged any regions of the source image that are of a similar intensity to the target. While this is beneficial in medical applications (removing this term in earlier experiments caused inappropriate modifications to the background), it may suppress the network’s efforts to enhance the foreground ROIs. Further work is needed to isolate the effect of the identity loss on contrast enhancement and to find a suitable replacement if necessary.

UNet-Phase and Pix2Pix both over-enhanced tumour in both phases (Pix2Pix was significantly more biased on pairwise ANCOVA intercept comparison). Pix2Pix also over-enhanced medulla in both phases ($p < 0.001$, Table 2) and generated the brightest ROI intensities of all networks ($p < 0.001$, Figs. 2 and 3). Visually, Pix2Pix generates greater enhancement (most apparent in the kidney—see red arrows in Fig. 4) than the other techniques, in line with its tendency towards over-enhancement, while U-Net provides more realistic intensities, particularly in the kidneys. The impact of “over-enhancing” certain regions is not necessarily problematic depending on the use case, for instance, in identifying vasculature and relevant anatomy.

### Task-based evaluation

There was no significant difference in Dice scores (Table 3) on H-test when the segmentation networks were trained on either NGE ground truth or the predicted NGE images. The CME phase did reach significance; on pairwise testing, there was no difference in performance between any generated images and CME ground truth, but UNet-Phase and CycleGAN did outperform Pix2Pix ($p < 0.01$). Both enhanced the tumour significantly less than Pix2Pix as well as having no bias in the medulla, indicating that quantitatively realistic ROI intensities are an important factor for downstream tasks of this nature.

### Proportional bias

The most obvious finding in Fig. 2 is that the ranges of ground truth intensities are wider than that of the more consistent proposed methods. The data demonstrate a statistically significant proportional bias for all models in aorta, cortex and medulla for CME predictions, i.e. their bias changes with intensity in these brightly enhancing regions. None of these proportional biases were significantly different on pairwise regression slope comparison.

The effect of the proportional bias in the highly enhancing CME ROIs is unclear; the generated intensities are physically and visually realistic, and it is likely that this is due to the larger inter-subject variation in ground truth intensity compared to the more consistent enhancement by the proposed methods, but it would benefit from further scrutiny in a human readability study. As well as showing us that each network and ROI has a different bias and must therefore be calibrated individually, an additional benefit of Bland–Altman analysis is that this information can be used to perform calibration in a future automated pipeline by removing any constant bias from a given ROI’s predicted intensities.

### Peri-procedural contrast enhancement

**Fig. 6:**
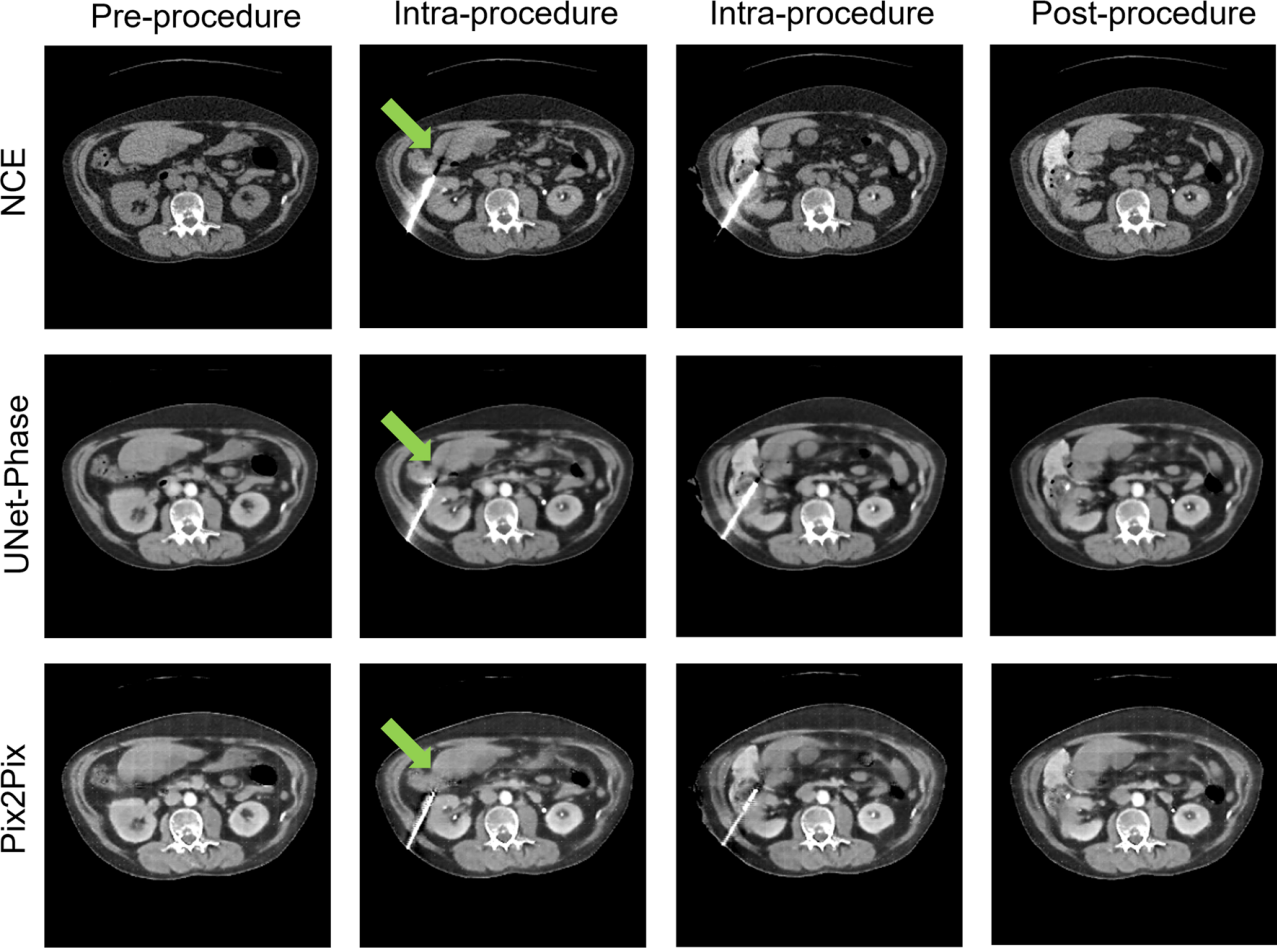
Images from before, during and after procedure. Green arrows: in-painting of photon starvation artefact

**Fig. 7:**
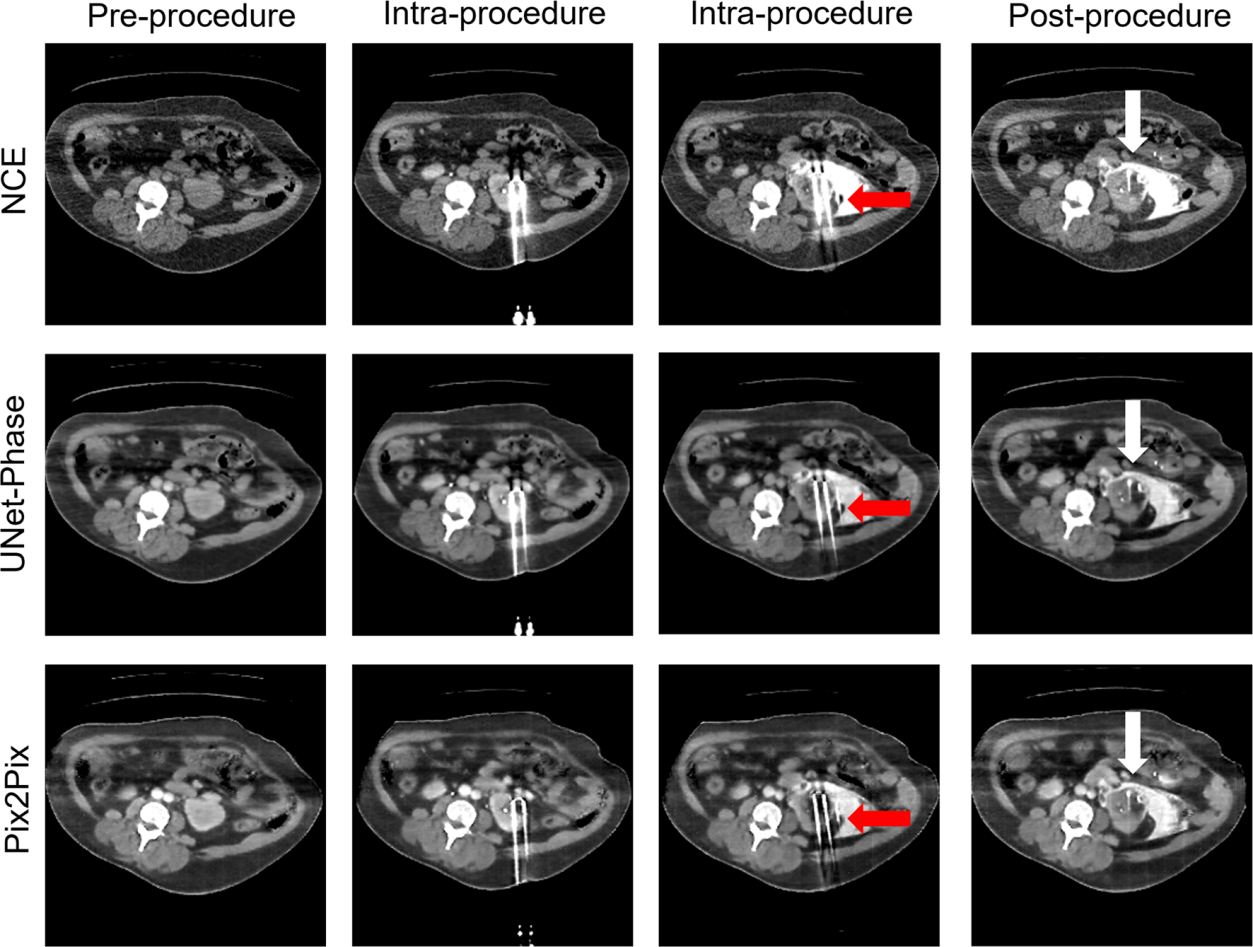
Images from before, during and after procedure, white arrows: improved visualisation of tumour, red arrows: improved visualisation of needles

Lastly, we examine the use of UNet-Phase and Pix2Pix with images taken during the procedure (i.e. with needles and hydro-dissection contrast in view) that were not present in the training dataset. Two examples are shown in Figs. 6 and 7 for the pre-procedural NCE image, two intra-procedural images and lastly one post-procedural image. Figure 6 confirms the networks’ ability to enhance aorta and kidney ROIs (CME phase) in images containing features they have not encountered before (needle in columns 2 and 3, and hydro-dissection in columns 3 and 4).

Figure 7 shows a case where UNet-Phase struggles to enhance the aorta correctly, and Pix2Pix erroneously enhances the vena cava (potentially due to the proximity of previously unseen features). However, we see that in the post-procedural images, the tumour is higher contrast with lower intensity values, making it more visible for both networks (white arrow) than in the un-enhanced image.

Interestingly, Pix2Pix in-paints the photon starvation artefact from the needle (Fig. 6, green arrow, also seen in Fig. 7), while both networks (UNet-Phase to a lesser extent) also reduce the streaking around the needle. It is unclear why needle artefact removal and improved tumour visualisation occurred—it is possible that the networks are generalising sCE to other high contrast regions—but this suggests a potentially useful future research direction for combining sCE and image quality enhancement for interventional procedures.

### Limitations

Contrast agents are used to identify clinically relevant features by enhancing the feature (e.g. a tumour) more or less than the surrounding tissue. However, all networks enhanced the tumour and surrounding tissue to a similar degree (white arrows, Figs. 4 and 5), limiting the amount of contrast that can be used to identify the tumour. The contrast enhancement patterns are generated by patient-specific characteristics that may not be apparent in a dataset of this size, or may exist below the scanner resolution—a more detailed study with additional data would be needed to address this. Despite this limitation, performance when segmenting the tumours from these predicted images appeared unaffected (aside from Pix2Pix for CME as stated above).

## Conclusion

In this paper, we proposed a framework for multi-phase synthetic contrast enhancement, as well as investigating the effect of a supervisory signal on model performance. The results show that our framework matches the performance of separately trained CNNs in all ROIs and exceeds it in the kidneys. In addition, the CycleGAN lacking any form of input from the ground truth intensities performed worst, while the Pix2Pix (adversarial with supervision) generated the most intense ROIs and the fully supervised U-Net was most quantitatively accurate. The proposed techniques generate more consistent enhancement than seen in the ground truth images, while assessing the bias by region and phase can inform usage of models with different characteristics for different tasks—UNet-Phase, with its smaller bias, outperforms Pix2Pix for segmentation, while Pix2Pix generates more vivid vasculature and renal medulla. Lastly, we have shown that UNet-Phase and Pix2Pix can generalise to unseen intra-procedural data, a more challenging task given the addition of needles, hydro-dissection contrast and the movement of organs over time. Crucially, the models also improve needle artefacts and tumour visualisation, indicating potential for multi-task sCE and image quality applications.

## Data Availability

Not applicable

## Declarations

### Code availability

https://github.com/markpinnock/synthetic-contrast-enhancement/tree/main/multi-phase-sce..

## References

[1] Mues AC, Landman J (2010). Results of kidney tumor cryoablation: renal function preservation and oncologic efficacy. World J Urol.

[2] Uppot RN, Silverman SG, Zagoria RJ, Tuncali K, Childs DD, Gervais DA (2009). Imaging-guided percutaneous ablation of renal cell carcinoma: a primer of how we do it. Am J Roentgenol.

[3] Permpongkosol S, Nielsen ME, Solomon SB (2006). Percutaneous renal cryoablation. Urology.

[4] Sahbaee P, Abadi E, Segars WP, Marin D, Nelson RC, Samei E (2017) The effect of contrast material on radiation dose at CT: Part II. A systematic evaluation across 58 patient models. Radiology 283(3), 749–757 10.1148/radiol.2017152852 PMC5452877 28287916

[5] Bottinor W, Polkampally P, Jovin I (2013). Adverse reactions to iodinated contrast media. Int J Angiol.

[6] Hirsch JD, Siegel EL, Balasubramanian S, Wang KC (2015) We built this house it’s time to move in: leveraging existing DICOM structure to more completely utilize readily available detailed contrast administration information. J Digit Imaging 28(4), 407–411 10.1007/s10278-015-9771-y PMC4501949 25700615

[7] Al-Ameen Z, Sulong G, Rehman A, Al-Dhelaan A, Saba T, Al-Rodhaan M (2015). An innovative technique for contrast enhancement of computed tomography images using normalized gamma-corrected contrast-limited adaptive histogram equalization. EURASIP J Adv Signal Process.

[8] Bitter I, Van Uitert R, Wolf I, Tzatha E, Gharib AM, Summers R, Meinzer HP, Pettigrew R (2006) Virtual contrast for coronary vessels based on level set generated subvoxel accurate centerlines. Int J Biomed Imaging 10.1155/IJBI/2006/94025 PMC2324051 23165062

[9] Mukherjee S, Acton ST (2015) Oriented filters for vessel contrast enhancement with local directional evidence. In: IEEE 12th international symposium on biomedical imaging (ISBI), vol 2015-July, pp 503–506. IEEE Computer Society

[10] Gong E, Pauly JM, Wintermark M, Zaharchuk G (2018). Deep learning enables reduced gadolinium dose for contrast-enhanced brain MRI. J Magn Reson Imaging.

[11] Kleesiek J, Morshuis JN, Isensee F, Deike-Hofmann K, Paech D, Kickingereder P, Köthe U, Rother C, Forsting M, Wick W, Bendszus M, Schlemmer H-P, Radbruch A (2019). Can virtual contrast enhancement in brain MRI replace gadolinium? A feasibility study. Invest Radiol.

[12] Montalt-Tordera J, Quail M, Steeden JA, Muthurangu V (2021). Reducing contrast agent dose in cardiovascular MR angiography with deep learning. J Magn Reson Imaging.

[13] Zheng Z, Ma L, Yang S, Boumaraf S, Liu X, Ma X (2021) U-SDRC: a novel deep learning-based method for lesion enhancement in liver CT images. In: Landman BA, Išgum I (eds) Medical imaging 2021: image processing, vol 11596, p 92. SPIE

[14] Sumida I, Magome T, Kitamori H, Das IJ, Yamaguchi H, Kizaki H, Aboshi K, Yamashita K, Yamada Y, Seo Y, Isohashi F, Ogawa K (2019). Deep convolutional neural network for reduction of contrast-enhanced region on CT images. J Radiat Res.

[15] Li Y, Li K, Garrett J, Chen G-H (2021) Generation of virtual non-contrast (VNC) image from dual energy CT scans using deep learning. In: Bosmans H, Zhao W, Yu L (eds) Medical imaging 2021: physics of medical imaging, vol 2021, p 48. SPIE

[16] Sandfort V, Yan K, Pickhardt PJ, Summers RM (2019) Data augmentation using generative adversarial networks (CycleGAN) to improve generalizability in CT segmentation tasks. Sci Rep 9(1) 10.1038/s41598-019-52737-x PMC6858365 31729403

[17] Bustamante M, Viola F, Carlhäll C-J, Ebbers T (2021). Using deep learning to emulate the use of an external contrast agent in cardiovascular 4D flow MRI. J Magn Reson Imaging.

[18] Choi JW, Cho YJ, Ha JY, Lee SB, Lee S, Choi YH, Cheon JE, Kim WS (2021). Generating synthetic contrast enhancement from non-contrast chest computed tomography using a generative adversarial network. Sci Rep.

[19] Kim SW, Kim JH, Kwak S, Seo M, Ryoo C, Shin CI, Jang S, Cho J, Kim YH, Jeon K (2021). The feasibility of deep learning-based synthetic contrast-enhanced CT from nonenhanced CT in emergency department patients with acute abdominal pain. Sci Rep.

[20] Liu J, Tian Y, Ağıldere AM, Haberal KM, Coşkun M, Duzgol C, Akin O (2020) DyeFreeNet: deep virtual contrast CT synthesis. In: Simulation and synthesis in medical imaging, vol 12417 LNCS, pp 80–89. Springer

[21] Chandrashekar A, Shivakumar N, Lapolla P, Handa A, Grau V, Lee R (2020) A deep learning approach to generate contrast-enhanced computerised tomography angiograms without the use of intravenous contrast agents. Eur Heart J 41(Suppl 2)

[22] Xie H, Lei Y, Wang T, Patel P, Curran WJ, Liu T, Tang X, Yang X (2021) Generation of contrast-enhanced CT with residual cycle-consistent generative adversarial network (Res-CycleGAN). In: Bosman, H, Zhao W, Yu L (eds) Medical imaging 2021: physics of medical imaging, vol 11595, p 141. SPIE-Intl Soc Optical Eng

[23] Seager M, Kumar S, Lim E, Munneke G, Bandula S, Walkden M (2020). Renal cryoablation: a practical guide for interventional radiologists. Br J Radiol.

[24] Yu H, Liu D, Shi H, Yu H, Wang Z, Wang X, Cross B, Bramlet M, Huang TS (2017) Computed tomography super-resolution using convolutional neural networks. In: IEEE international conference on image processing (ICIP), pp 3944–3948

[25] Isola P, Zhu J-Y, Zhou T, Efros AA (2017) Image-to-image translation with conditional adversarial networks. In: Proceedings of IEEE conference on computer vision and pattern recognition

[26] Zhu JY, Park T, Isola P, Efros AA (2017) Unpaired Image-to-Image Translation Using Cycle-Consistent Adversarial Networks. In: Proceedings of the IEEE international conference on computer vision, vol 2017, pp 2242–2251. Institute of Electrical and Electronics Engineers Inc

[27] Cohen JP, Luck M, Honari S (2018) Distribution matching losses can hallucinate features in medical image translation. Lecture notes in computer science (including subseries Lecture Notes in Artificial Intelligence and Lecture Notes in Bioinformatics) 11070 LNCS, pp 529–536

[28] Ronneberger O, Fischer P, Brox T (2015) U-Net: convolutional networks for biomedical image segmentation. In: International conference on medical image computing and computer-assisted intervention, vol LNCS 9351, pp 234–241. Springer

[29] Çiçek Ö, Abdulkadir A, Lienkamp SS, Brox T, Ronneberger O (2016) 3D U-Net: learning dense volumetric segmentation from sparse annotation. In: International conference on medical image computing and computer-assisted intervention, vol. 9901 LNCS, pp 424–432. Springer

[30] Ulyanov D, Vedaldi A, Lempitsky V (2016) Instance Normalization: The Missing Ingredient for Fast Stylization. arXiv preprint arXiv:1607.08022

[31] Bland JM, Altman DG (1999). Measuring agreement in method comparison studies. Stat Methods Med Res.

[32] Poline JB, Brett M (2012). The general linear model and fMRI: Does love last forever?. Neuroimage.

[33] Fedorov A, Beichel R, Kalpathy-Cramer J, Finet J, Fillion-Robin JC, Pujol S, Bauer C, Jennings D, Fennessy F, Sonka M, Buatti J, Aylward S, Miller JV, Pieper S, Kikinis R (2012). 3D slicer as an image computing platform for the quantitative imaging network. Magn Reson Imaging.

[34] Huang F, Chien CL (2020) Patch-based painting style transfer. In: 2020 IEEE international conference on consumer electronics—Taiwan, ICCE-Taiwan 2020. Institute of Electrical and Electronics Engineers Inc

[35] Zhao S, Liu Z, Lin J, Zhu J-Y, Han S (2020) Differentiable augmentation for data-efficient GAN training. Adv Neural Inf Process Syst

